# Optical Ultrasound Generation and Detection for Intravascular Imaging: A Review

**DOI:** 10.1155/2018/3182483

**Published:** 2018-04-30

**Authors:** Tianrui Zhao, Lei Su, Wenfeng Xia

**Affiliations:** ^1^School of Engineering and Materials Science, Queen Mary University of London, London E1 4NS, UK; ^2^Wellcome/EPSRC Centre for Interventional and Surgical Sciences, University College London, Charles Bell House, 67-73 Riding House Street, London W1W 7EJ, UK; ^3^Department of Medical Physics and Biomedical Engineering, University College London, Gower Street, London WC1E 6BT, UK

## Abstract

Combined ultrasound and photoacoustic imaging has attracted significant interests for intravascular imaging such as atheromatous plaque detection, with ultrasound imaging providing spatial location and morphology and photoacoustic imaging highlighting molecular composition of the plaque. Conventional ultrasound imaging systems utilize piezoelectric ultrasound transducers, which suffer from limited frequency bandwidths and reduced sensitivity with miniature transducer elements. Recent advances on optical methods for both ultrasound generation and detection have shown great promise, as they provide efficient and ultrabroadband ultrasound generation and sensitive and ultrabroadband ultrasound detection. As such, all-optical ultrasound imaging has a great potential to become a next generation ultrasound imaging method. In this paper, we review recent developments on optical ultrasound transmitters, detectors, and all-optical ultrasound imaging systems, with a particular focus on fiber-based probes for intravascular imaging. We further discuss our thoughts on future directions on developing combined all-optical photoacoustic and ultrasound imaging systems for intravascular imaging.

## 1. Introduction

Intravascular imaging is an invasive approach that acquires images of diseased blood vessels, providing detailed and accurate measurements of pathology information. For example, atherosclerosis, a chief cause of cardiovascular disease, is an arterial disease attributed to build up of fatty material on the inner walls of arteries [1, 2]. Atheromatous plaque is a raised area on arterial walls and mainly comprised of lipids, calcium, fibrous tissues, and macrophage cells. The atheromatous plaque accumulates on the interior vessel wall, causing stenosis (narrowing) of vessels and obstructing blood flow [2]. Angiography is the conventional imaging method used for visualization of blood vessels, while it is problematic to obtain crucial information about atheromatous plaques without imaging internal wall of blood vessels [3].

To address this problem, intravascular ultrasound (IVUS) has been used as a complementary imaging modality to angiography to provide structure information of coronary arteries with high spatial resolution (70–200 *μ*m). This information includes lumen and vessel dimensions, plaque morphology, and location and thus enables the determination of the degree of stenosis [4]. However, the vulnerability of the plaques is not determined by the degree of stenosis but the composition of the plaques [4]. Thus, an imaging method that can differentiate plaque composition is highly desired for accuracy diagnose. 

**Figure 1:**
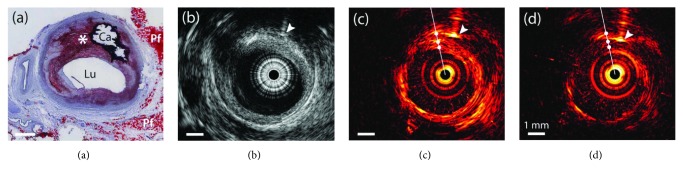
Examples of IVPA and IVUS imaging results of an advanced human atherosclerotic plaque. (a) Histology of the plaque with a calcified area (Ca), periadventitial fat (Pf), and a lipid-rich plaque (^∗^). (b) Ultrasound image. (c) Photoacoustic images with excitation light at 1210 nm and (d) 1230 nm. Reproduced with permission from [12], Copyright 2011, The Optical Society.

Recent studies showed that intravascular photoacoustic imaging (IVPA) has the potential to characterize plaque types [5, 6]. Photoacoustic imaging is based on the photoacoustic effect: upon pulsed laser excitation, tissues absorb light and results in a local temperature rise, causing rapid thermal expansion of tissues that generates acoustic waves as photoacoustic signals [7]. These acoustic waves can be detected by ultrasound receivers and images showing the distribution optical absorption of chromophores can be reconstructed by spatially resolving these signals. The strengths of photoacoustic signals are mainly dictated by local optical absorption coefficient of the tissue and the fluence of the local light. As different plaques show varying absorption coefficient for the same incident laser [7], they generate photoacoustic signals with varying pressures, so their composition can be characterized in photoacoustic images. In addition to the improvement of tissue identification, photoacoustic imaging at multiple wavelengths resolves the concentrations of specific absorbers [8]. Recently, the advancement in IVPA imaging speed paved the way for its clinical translation [9–11]. However, PA imaging suffers from relatively low imaging depth due to rapid reduction of light fluence with tissue depth [7], which limits its application on imaging intact plaque morphology and artery wall. Typically, an IVPA catheter comprises of fiber optics for light delivery and an ultrasound transducer to receive PA signals. Meanwhile, the transducer transmits ultrasound signals and hence pulse-echo ultrasound imaging can be combined in the same device [5, 6]. This design offers both advantages of IVUS (deep penetration) and IVPA (composite contrast). Recently, several endoscopic intravascular imaging systems were developed that combine both ultrasound and photoacoustic imaging [12–20]. An example of imaging results is shown in Figure 1. The morphology of an advanced human atherosclerotic plaque was confirmed in ultrasound image, while photoacoustic images highlighted the periadventitial fat and eccentric plaque, demonstrating that photoacoustic imaging can provide complementary information to conventional ultrasound imaging with tissue-type contrast [12].

Piezoelectric materials are conventionally used as the ultrasound transducers in IVPA imaging catheters. In order to achieve high sensitivity of IVPA, a transducer with low frequency bandwidth < 8 MHz is required while high frequency bandwidth > 20 MHz is preferred for high-resolution IVUS imaging [6]. Dual-element transducers were explored to meet the requirements of both sensitive IVPA and high-resolution IVUS imaging [21, 22]; however, it increased the complexity and the diameter of the combined IVUS/IVPA catheter. Polyvinylidene difluoride (PVDF) transducer with frequency in 2–15 MHz was explored for IVPA imaging [23], while higher frequency is significant for improving imaging resolution. Furthermore, optically opaque piezoelectric transducers are difficult to integrate with other imaging devices. As an alternative, optical methods to generate and receive ultrasound have been developed recently, which provides promise for the fabrication of miniature IVUS/IVPA imaging device with high sensitivity and resolution. Several studies have pointed to composites consisting of optically absorbing and elastomeric components as particularly efficient for high-frequency ultrasound generation [24–34]. Upon the excitation of incident laser, the absorbers convert optical energy into the local temperature rise, which generates acoustic waves following the laser profile. Polydimethylsiloxane (PDMS) has been highlighted as a promising elastomeric component for optical ultrasound generation as it is biocompatible and has a high thermal expansion coefficient [33]. Optical absorbers for ultrasound generation that have been studied include carbonaceous materials such as carbon black [24, 25], graphite [26], graphene [27], carbon nanotubes (CNTs) [28–30], and carbon nanofibers [31] as well as gold nanoparticles (AuNPs) [32–35]. On the other hand, several optic fiber-based detectors, such as Fabry-Perot (FP) etalons [36–39], microring resonators [40, 41], and fiber Bragg gratings (FBGs) [42, 43], were developed to receive ultrasound waves. These detectors mainly measure the acoustically caused deformation. By recording the frequency or power change caused by deformation of the optics, ultrasound pressure can be measured with high sensitivity and wide frequency bandwidth. 

All-optical ultrasound imaging systems, which comprise of optical ultrasound transmitters and detectors, have been realized with demonstrated tissue imaging capability [44–48]. These systems show four main advantages over the present ones: First, due to the fiber-based nature, these imaging devices can be readily miniaturized (<1 mm in diameter). Second, in contrast to piezoelectric elements, optical ultrasound transmitters and detectors possess high ultrasound transmission efficiency and sensitivity, respectively, even with miniature sizes. Third, the compatibility with magnetic resonance imaging (MRI) makes all-optical imaging devices applicable in many clinical procedures where intraoperative MRI is used. Finally, all-optical ultrasound transmitters and receivers facilitate the integration of both ultrasound and photoacoustic imaging, where a single optical fiber could be used to both deliver the photoacoustic excitation light and transmit ultrasound. AuNPs, which are widely used as contrast agent in photoacoustic imaging and exhibit strong absorption [49–53], were developed to achieve this dual-modality transmission [33].

In this paper, we reviewed recent developments on optical ultrasound transmitters, detectors, and their applications in all-optical ultrasound imaging systems, primarily focusing on their current or potential applications in intravascular imaging. Recent studies on combined IVUS and IVPA probes are also presented, and further directions of the all-optical ultrasound imaging systems for hybrid IVUS/IVPA imaging are discussed.

## 2. Optical Ultrasound Generation

Optical ultrasound transmitters are based on photoacoustic effect while it is designed for echo-pulse ultrasound imaging. Different from the photoacoustic imaging where photoacoustic signals are excited from target tissues, in optical ultrasound generation, ultrasound waves are generated from an optically absorbing coating, and sent to the target tissues for pulse-echo ultrasound imaging. Under the pulsed laser excitation, the coating absorbs absorb optical energy and convert the energy to rapid temperature rise, which results in ultrasound generation. Composite coatings with strong optical absorption and high thermal expansion coefficient are desirable for maximizing the ultrasound signals. Carbon materials [24–31], with strong optical absorption and great thermal conductivity, have been developed as laser absorbers for optical ultrasound generation. Besides carbon materials, metal nanoparticles such as AuNPs [32–35] have also been investigated. Due to the narrow but strong absorption band, AuNPs are promising to be used for the integration of IVUS and IVPA. Polydimethylsiloxane (PDMS) was highlighted as an elastomeric component with high thermal expansion coefficient and similar acoustic impedance to tissues [54–57]. We refer readers to [33] for PDMS composites reviewed according to different fabrication strategies. In this section, composites are reviewed according to different structures, such as planar and fiber-based design. Factors influencing the performance of ultrasound generation are discussed. Performance characteristics of both planar and fiber-based optical ultrasound transmitters are summarised in Tables 1 and 2, respectively.

### 2.1. Planar Transmitters

A series of composites comprising of optical absorbers and thermal elastic materials were deposited onto glass substrates for generating ultrasound optically [24–28]. Spin-coating was highlighted to produce both polymer and polymer-based composite thin films onto substrates. By adjusting spinning speed and duration, the thickness of thin films can be controlled at micrometer level [28]. Through the spinning approach, multilayer structure coatings were obtained with improved ultrasound generation performance than single-layer ones.

**Figure 2:**
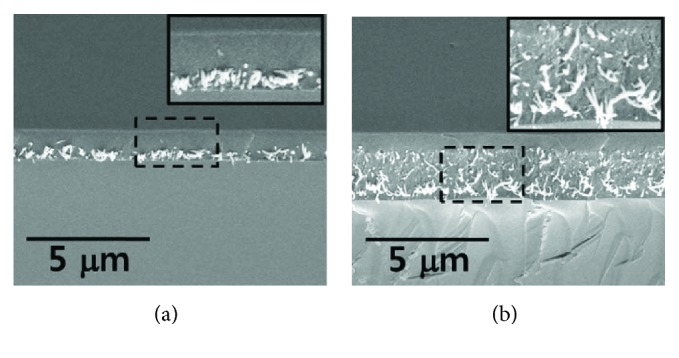
SEM images of multilayer CNTs-PDMS composite films with CNT growth time of 1 min in (a) and 3 min in (b). Reproduced with permission from [28], Copyright 2010, AIP Publishing.

Thin metallic coatings on solid substrates generate ultrasound under pulsed or modulated light excitation due to their strong optical absorption. However, metal thin films suffer from low thermal expansion coefficients and considerable proportion of the incident radiation is reflected from the metal surface, leading to low pressure of the generated acoustic waves. In recent studies, Cr and Al thin films were mainly used as reference to other absorptive materials [24, 27]. A composite film was fabricated by spin-coating a mixture of carbon black, PDMS, and toluene onto a microscope glass slide, which improved the ultrasound strength by appropriately 20 dB compared with a reference Cr film [24]. However, the film was so thick that the generated ultrasound was attenuated within the outer layer film. The attenuation was especially obvious at high frequency region (>50 MHz), which is significant for high-resolution imaging. To solve this issue, a thinner black PDMS thin film using pure PDMS and larger carbon black particle mixture was fabricated by improving spinning speed, improving the strength of ultrasound by nearly 10 dB [25]. However, this transmitter showed similar performance in high-frequency region. As an alternative, CNTs were used to for high-frequency ultrasound generation [28]. CNTs suffer the problem of agglomeration in polymer and hence cause different absorbance in different areas. In order to solve the problem, a multilayer structure was designed with CNTs sandwiched by a transparent substrate and a PDMS layer (Figure 2). With CNTs grown on a glass substrate and coated with a layer of PDMS, the transmitter generated ultrasound signals 25 dB stronger than that in the Cr reference, while the corresponding bandwidth was calculated to be more than 120 MHz at −10 dB level [28].

**Figure 3:**
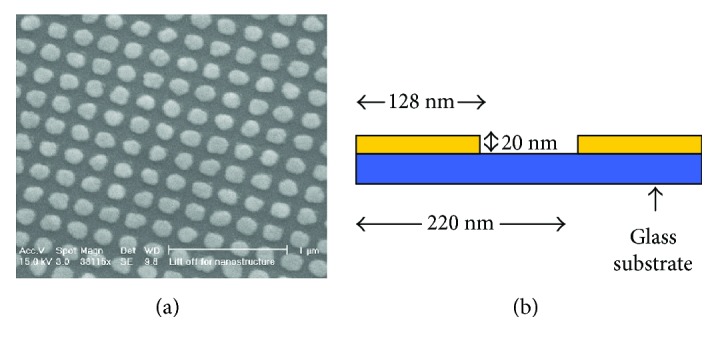
(a) SEM images of the AuNP array. (b) Sketch of the side view of AuNP array on a glass substrate. Reproduced with permission from [32], Copyright 2006, AIP Publishing.

There are three reasons for CNTs to be efficient for high-frequency ultrasound generation: Firstly, the nanoscale dimension and hollow cylinder structure inherently allow rapid heat transition to the surrounding medium in the order of nanoseconds. Secondly, CNTs exhibit excellent thermal conductivity (20–30 times higher than that of typical metal) and thus allow the generation of strong acoustic pressure [58–60]. Finally, CNTs thin film can be grown onto substrates by chemical methods, enabling the uniform distribution of CNTs, which enables that the ultrasound waves are evenly generated throughout the composite film. In addition, CNTs were also coated onto concave lens to generate tight and strong ultrasound [61]. Other carbon nanomaterials such as carbon nanofibers (CNFs) [31] and reduced graphene oxide (rGO) [27] were also explored. CNFs were used as optical absorber in the same structure as CNTs, improving the maximum acoustic pressure by 17.62 dB compared to carbon black-PDMS films [28]. In a recent study by Lee et al., a rGo thin film was sandwiched by a glass substrate and a carbon black-PDMS film to fabricate the ultrasound transmitter, which generated ultrasound waves with the pressure 76 times higher than Al thin film [27]. Besides carbon materials, metallic particles with nanostructure have also been used to optically generate ultrasound as the optically absorbing layer. In a study by Hou et al., a stucture comprising of 2D AuNP array, which was sandwiched between a transparent substrate and a 4.5 *μ*m thick PDMS layer, was fabricated using a 2D nanosquare array mold (Figure 3) [32]. This transmitter produced ultrasound signal with strong strength in high-frequency range (50–100 MHz). A mixture of gold salt with PDMS was also coated onto glass substrates for ultrasound generation, with varying gold salt concentration and thickness leading to different photoacoustic generation efficiency [34].

### 2.2. Fiber-Based Transmitters

Miniature transmitters could be extremely useful in highly limited space to facilitate specific applications such as IVUS and IVPA imaging. Materials used in planar transmitter were also used for fiber-based transducer fabrication. In contrast to the planar substrate, an optical fiber provides restricted area for coatings, which limits the pressure of the generated ultrasound. Recently, with the development of highly efficient laser absorptive materials such as CNTs and AuNPs, strong ultrasound waves have been generated.

**Figure 4:**
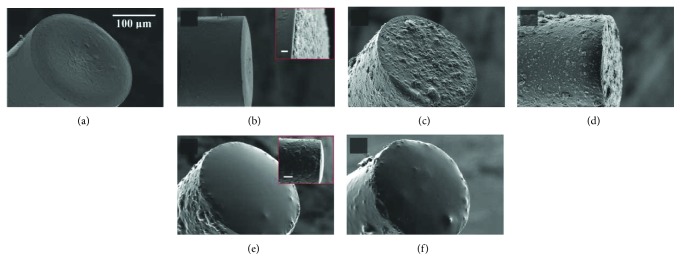
SEM images of CNTs-PDMS-coated optical fibers using MWCNT-xylene in (a) and (a), inset: side-view, scalebar: 1 *μ*m, MWCNT-gel in (c) and (d). (e) MWCNT-gel/PDMS-coated fiber end (inset, side-view, scale bar: 50 *μ*m). (f) MWCNT-PDMS-coated fiber end. Reproduced with permission from [30], Copyright 2016, John Wiley and Sons.

As an alternative to spin-coating on planar substrates, dip-coating was implemented to fabricate thin composite film at optical fiber ends [29, 30]. In this method, cleaved optical fibers were dipped into uncured composite, after which the cured composite coatings were used for ultrasound generation. As it is challenging to grow CNTs on fiber ends, a strategy involved CNT functionalization [29] was studied to dissolve CNTs into xylene and the CNT/xylene solution was mixed with PDMS solution. With pulsed light illumination, comparable signals to that generated by piezoelectric transducers were achieved. To achieve higher frequency ultrasound, Noimark et al. used a multilayer CNTs-PDMS coating on the optical fiber. An oleylamine-functionalized pyrene ligand was used to functionalize MWCNTs, and the resulting gel solution was dip-coated onto optical fibers (maximum coating thickness of less than 1 *μ*m). Then the fiber end was dipped into uncured PDMS to coat the thermal elastic layer (Figure 4). This optical ultrasound transmitter generated strong ultrasound signal with pressures of up to 21.5 MPa with corresponding bandwidths of around 39.8 MHz [30], which is the largest ultrasound pressure achieved using optical fiber-based transmitters. AuNPs were also developed to fabricate miniature transmitters. An in situ strategy has been explored to develop AuNPs-PDMS composites, in which the mixture of PDMS and gold salt was dip-coated onto fiber ends [35]. The in situ reduction of gold salt within the PDMS produced AuNPs-PDMS composite. AuNPs were also patterned on the end face of an optical fiber using a focused ion beam technique and used for ultrasound generation. Different from carbon materials, AuNPs exhibit narrow but strong optical absorption peak at specific wavelength, with negligible absorption at other wavelengths. This property comes from surface plasmon resonance effect [48, 50]. When changing the laser wavelength to low-absorptive region, laser is delivered through the AuNPs-PDMS composite to excite photoacoustic signals from the imaging target. Based on this specific property, a dual transmitter was fabricated and performed hybrid ultrasound and photoacoustic imaging [33]. Dye was also used for the hybrid signal generation through one optical fiber [33].

On optical fibers, some side-view ultrasound transmitters were achieved, mainly by coating laser absorptive materials on the sidewall of the optical fibers. A part of fiber cladding was removed and refilled with graphite-epoxy composite [62]. A ghost mode of a tilted fiber Bragg grating and graphite/epoxy mixture was used to generate side-view ultrasound [63]. Although ultrasound signals were obtained from these transmitters, the signal strengths were limited to be used for ultrasound imaging. This is mainly due to the low laser flux on side. In future study, effective methods to delivery laser to side views are required.

To sum up, ultrasound signals with comparable intensity and bandwidth to piezoelectric transducer were generated by optical transmitters. As stronger ultrasound signal allows larger penetration depth and high-frequency ultrasound offers high resolution in resulting images, optical ultrasound transmitters were used for pulse-echo ultrasound imaging, which will be discussed in Section 4.

Among light-absorptive materials, nanoscale materials showed advantages due to high optical absorption and rapid heat conduction, with CNTs and AuNPs emerging as excellent examples. Although CNTs generated the strongest ultrasound pressure, AuNPs have unique advantages for combining IVUS and IVPA due to their specific absorption for both ultrasound and laser transmissions.

The generated ultrasound frequency bandwidth is determined by the thickness of the composite film due to the rapid attenuation of the high frequency ultrasound components by the composite film. Spin-coating is an effective method to fabricate thin film on planar substrates with controlled thickness at micrometer level, while dip-coating remained the only method to coat optical fiber ends. However, this method involves the challenge to control film thickness, which is roughly controlled by dipping speed. In future study, micro fabrication process such as 3D printing and CVD can be explored to precisely coat composite films. Furthermore, coatings dipped onto the fiber ends showed fixed structure (dome-like) [29, 30], which generated a corresponding acoustic field. Specially shaped coating, such as a concave surface, is promising to focus acoustic waves to a desirable point, which narrows the ultrasound beam and hence improves the lateral resolution.

The structure of composite coatings also showed influence on resulting ultrasound, mainly associated with the attenuation in the high frequency. Generally, ultrasound signals achieved from multilayer transmitters showed larger bandwidth. It is likely that laser absorptive materials only transfer heat to the PDMS layer in multilayer structures, avoiding attenuating the acoustic waves by absorbers. There has not been a study to measure the thermal elastic coefficient change between PDMS and PDMS-based composites. Theoretically, the distribution of absorbers into PDMS changes the network of PDMS polymer chains, which may reduce the thermal expansion coefficient of PDMS and hence reduce the ultrasound pressure.

## 3. Optical Ultrasound Detection

Based on optical resonance, optical ultrasound detectors convert physical deformation caused by ultrasound into optical interference and hence record the acoustic signals. In contrast to piezoelectric detectors whose sensitivity falls off with decreasing element size, optical ones keep the sensitivity even when fabricated into hundreds of micrometers [36–43]. Furthermore, the flexibility and immunity to electromagnetic interference allow the application of optical detection with other diagnosis devices such as MRI. In this section, optical ultrasound detectors which have been or have potential to be used for intravascular ultrasound detection will be introduced, and the further development and applications will be discussed.

**Figure 5:**
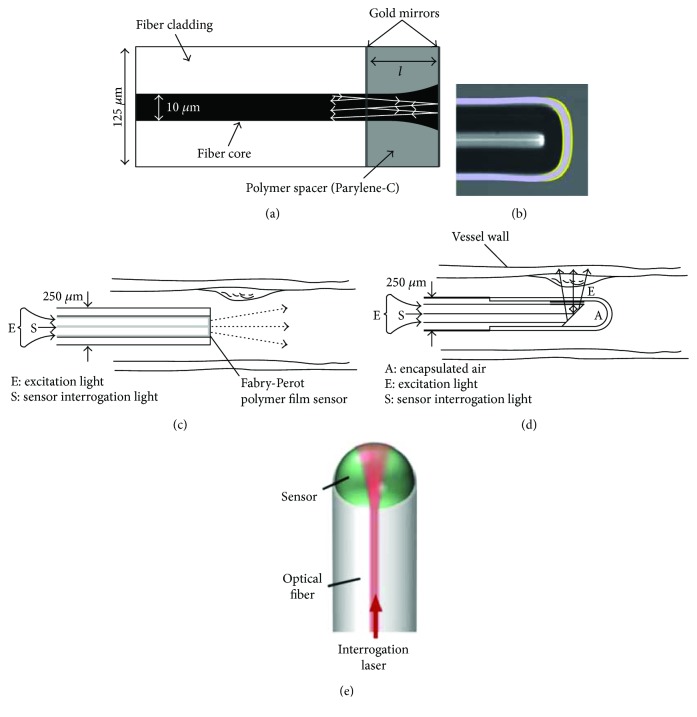
Major embodiments of fiber optic Fabry-Perot (FP) ultrasound detectors. (a) A schematic illustration of a FP sensor with a cylindric etalon cavity. (b) A gray scale microscopic image of a rounded-tip fiber with a concave etalon cavity. The design of endoscopic imaging probes with (c) forward-looking and (d) sideways-looking configurations. (e) The design of a highly sensitive planoconcave microresonator ultrasound sensor. Reproduced with permission, from [36] for (a); Copyright 2009, AIP Publishing, from [37] for (b); Copyright 2015, Zhang et al. from [38] for (c) and (d); and Copyright 2011, Zhang et al. from [39] for (e), Copyright 2017, Springer Nature.

Recently, several optical ultrasound detectors were developed with different structures, such as Fabry-Perot (FP) etalon [36–39], microring resonator [40, 41], and fiber Bragg grating (FBG) [42, 43]. The heart of the FP detector (Figure 5(a)) is a transparent film with two parallel reflecting surfaces (mirrors). This design reflects only light at a specific wavelength which is determined by the distance between two mirrors. In the detection process, a continuous interrogation laser was tuned to a wavelength at an edge of the FP cavity resonance. As ultrasound waves cause deformations of the transparent film and hence the distance between two mirrors, the intensities of the reflected laser light, which are linearly proportional to the acoustic pressure, are recovered. A series of FP sensors, both in planar or fiber-based designs, were fabricated for ultrasound and photoacoustic imaging [36–39, 64–68]. An example of fiber-based FP sensor is shown in Figure 5(a), with a Parylene-C spacer and two gold mirrors fabricated at the end of an optical fiber [36]. Double-cladding optical fibers with FP etalons were used for miniature photoacoustic imaging: gold mirrors were replaced by dichroic films to reflect the light in specific wavelength range. The excitation and interrogation lights are coupled into the different waveguides in the fiber at the same time. Both forward- and sideway-looking scanning probes were designed with different configurations (Figures 5(c) and 5(d)) [38]. The sensitivity of a FP etalon is determined by the sharpness of the resonance. In order to improve the sensitivity, concave geometry [37] and a planoconcave microresonator [39] were designed to improve the optical confinement between reflecting surfaces (Figures 5(b) and 5(e)). In addition to high sensitivity, the concave design also enables omnidirectional ultrasound detection.

Polymer microring resonators have also been studied for acoustic signal detection [40, 41]. In contrast to FP devices which depend on the deformation of a transparent film, the microring sensors use a coupled ring waveguide to form an optical cavity. The incoming ultrasound signal causes the deformation of the ring waveguide, inducing a corresponding shift of the resonant wavelength. By recording the corresponding optical intensity of the optical output, the corresponding acoustic signals can be recorded. Both minimal size and wide bandwidths were achieved in microring sensor [40, 41], providing the promise for invasive imaging. As the microring uses the light path outside the fiber, the optical fiber can independently deliver excitation laser for photoacoustic signal generation.

**Figure 6:**
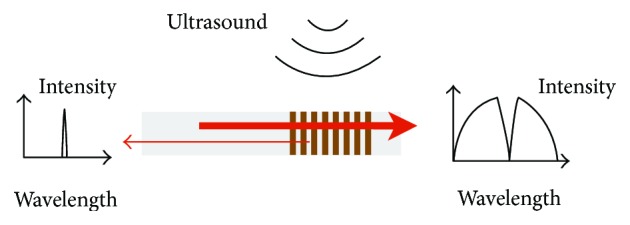
The principle for a FBG-based ultrasound detector. Coherent light at specific wavelength is reflected.

FBGs were also developed to detect acoustic signals by optical methods. The operation principle is shown in Figure 6. An acoustic wave causes perturbation on FBGs which leads to a shift in the FBG reflection spectrum. Using a narrow linewidth laser with wavelength locked to the middle-reflection wavelength of the spectrum, the variation of output power associated with the reflection shift and hence the ultrasound signals can be recorded. As the detection sensitivity is inherently determined by the spectral slopes, *π*-phase-shifted FBGs, which feature a sharp notch in the reflected spectrum, were primarily studied for high-frequency ultrasound detection [42, 43]. In recent research, the ultrasound signals were recorded with high pressure sensitivity and effective bandwidth [42].

In current imaging systems, both FP and microring sensors require a separated light waveguide and different laser sources for signal excitation and detection. Conceptually, a *π*-phase-shifted FBG can be considered as a FP cavity sensor. The difference is that a FP etalon depends on a dichroic film to separate excitation and interrogation lights. In contrast, FBGs separate laser into transmission and reflection parts from the same source, so a miniature photoacoustic imaging probe is promising using FBG sensor with only one laser source, which serves as excitation and interrogation light at the same time.

To sum up, optical ultrasound detectors showed high sensitivity and wide frequency band in ultrasound detection. Particularly, the planoconcave FP sensor provided excellent sensitivity with 0.093 kPa over 40 MHz [39]. The high sensitivity and wide ultrasound detection bandwidth allow optical ultrasound detector for both ultrasound and photoacoustic imaging. The all-optical imaging using optical ultrasound detectors will be discussed in Section 4. Performance characteristics of different fibre-optic ultrasound sensors are compared in Table 3.

## 4. All-Optical Ultrasound and Photoacoustic Imaging

**Figure 7:**
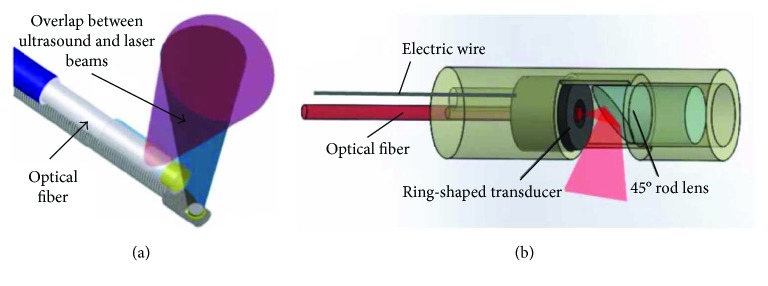
Examples of two main configurations of conventional IVPA catheters. (a) Ultrasound and laser beams overlap at the target position. (b) Collinear design allows both light and ultrasound propagation share the same path. Reproduced with permission from [13] for (a), Copyright 2010, AIP Publishing, and from [11] for (b), Copyright 2014, Springer Nature.

A combined IVUS/IVPA imaging device providing both vessel wall structure and molecular composition contrast of different tissues is highly desired. Conventionally, a IVPA catheter comprises of two necessary parts: a laser delivery path and an ultrasound transducer. Optical fibers with micromirrors or angle-polished ends are widely used to deliver laser beam in radial direction. Microsized piezoelectric transducers are conventionally used to transmit ultrasound and receive both reflected ultrasound and excited photoacoustic signals. Typically, there are two main IVPA configurations (Figure 7): In the first design, ultrasound is generated vertical or almost vertical to the optical fiber while the side-firing fiber is designed to deliver laser to overlap the ultrasound at a desired positon [5, 6]. The other type uses a collinear design to transmit both laser and ultrasound in the same path, exhibiting the advantage in sensitivity [18]. A series of IVUS/IVPA images were obtained; we refer the reader to [6] for IVPA imaging of vulnerable atherosclerotic plaque.

**Figure 8:**
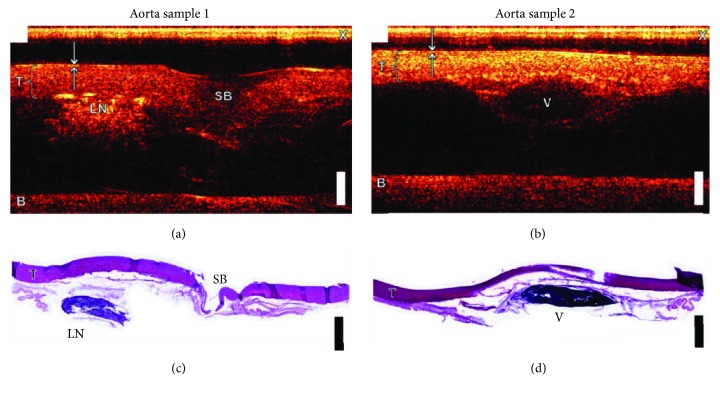
(a‐b) All-optical ultrasound images of swine aorta tissue using an optical fiber-based FP sensor. T, tunica media; X, cross-talk; B, the base of the tissue mount; SB, side branch; LD, lymph node; V, vessel. (c) and (d) Histological images of aorta sections corresponding to the ultrasound images in (a) and (b), respectively. Scale bar, 2 mm. Reproduced with permission from [45], Copyright 2015, The Optical Society.

However, conventional designs are limited for further minimization with constant or higher performance. This is mainly due to the piezoelectric ultrasound transducer, which suffers problems such as sensitivity and frequency bandwidth when fabricated into small sizes. All-optical ultrasound and photoacoustic imaging devices have been rapidly developed in the past decade, which is promising to be used for IVUS/IVPA imaging as an optical alternative. An all-optical ultrasound imaging device uses optical methods to generate and detect ultrasound signals. As photoacoustic signals are naturally acoustic waves, they can be detected by the same optical sensor. As mentioned before, CNTs-PDMS composite-coated optical fiber generated strong ultrasound signals [29] and FP etalons offer highly sensitive detection [36] for acoustic waves. An all-optical ultrasound imaging probe was fabricated using a fiber-based CNTs-PDMS transmitter and a FP sensor [45]. By translating the probe across the tissue sample, ultrasound images were achieved with high resolution and large penetration depth (Figure 8) [45]. Using the multilayer CNTs-PDMS transmitter, ultrasound images were achieved at the depth more than 10 mm [30]. With a microlens, pencil-beam all-optical ultrasound imaging was achieved and provided high-quality ultrasound images [46]. A fiber bundle was also used for all-optical ultrasound imaging with high resolution [47]. Recently, a planoconcave FP sensor which provides ultrahigh sensitivity was developed. Using the planoconcave sensor, a 3D ultrasound image with high resolution was obtained using a CNTs-PDMS-coated fiber as transmitter [40].

**Figure 9:**
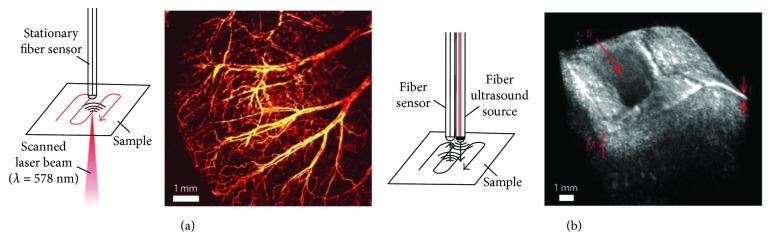
(a) Left: schematic illustration of an optical-resolution photoacoustic microscopy system using a highly sensitive fiber microresonator. Right: a representative high-resolution photoacoustic image of the mouse ear vasculature in vivo, demonstrating a large field-of-view. (b) Left: schematic illustration of an all-optical ultrasound imaging system using a highly sensitive fiber microresonator. Right: a representative 3D ultrasound image of ex vivo porcine aorta. Reproduced with permission from [39], Copyright 2017, Springer Nature.

The same planoconcave FP sensor was also used for photoacoustic imaging with an external laser source for ultrasound excitation. An optical resolution photoacoustic image was obtained [40]. The setup and images of ultrasound and photoacoustic imaging are shown in Figure 9. Different from this design, a typical all-optical IVPA device uses an optical fiber to deliver excitation laser and an optical sensor for ultrasound detection. As presented in Section 2, both forward and sideway all-optical IVPA probes were designed based on double-cladding fibers [38]. Besides FP etalons, fiber-based microring was also developed for all-optical photoacoustic imaging, with volumetric imaging of several phantoms achieved [41].

**Figure 10:**
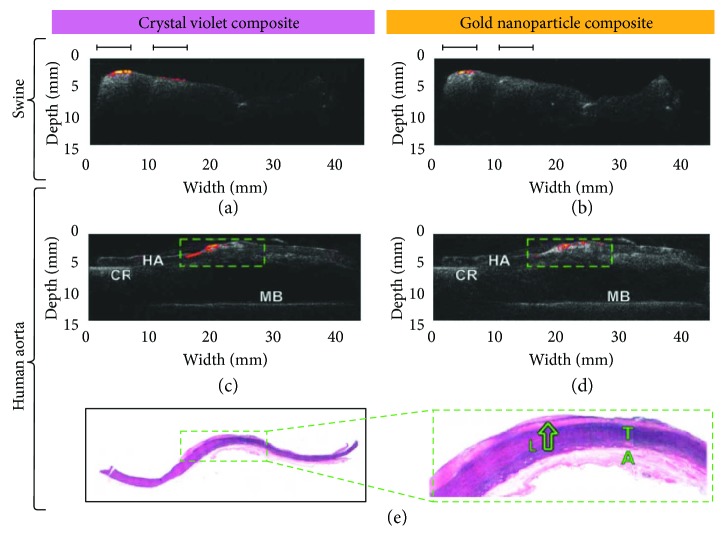
(a–d) Combined ultrasound and photoacoustic images achieved from the all-optical devices using crystal violet and AuNP composites for hybrid transmitters. Coregistered photoacoustic and ultrasound images were obtained from (a‐b) ex vivo swine abdominal tissue and (c‐d) human aorta. Colour-coded photoacoustic images were superimposed on the corresponding ultrasound images. The fatty regions are indicated with black bars. CR, cork ring; HA, human aorta tissue; MB, metal base. (e) Histological images of the imaged human aorta tissue. T, tunica media; A, adventitia. Reproduced with permission from [33], Copyright 2018, John Wiley and Sons.

**Figure 11:**
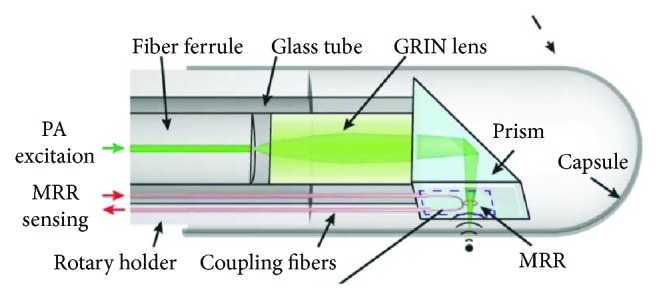
The design of the all-optical photoacoustic probe using a microring resonator. Reproduced with permission from [40]. Copyright 2014, The Optical Society.

Recently, probes using three optical fibers were fabricated for all-optical ultrasound and photoacoustic imaging [67, 68]. A Fabry-Perot cavity at the tip of a fiber offered acoustic detection, while a fiber delivered excitation laser and a carbon black-PDMS or CNTs-PDMS composite-coated fiber was used for ultrasound generation [67, 68]. Both ultrasound and photoacoustic images with high resolution and depth were achieved through these devices [67, 68]. As the optical transmitters use pulsed laser for ultrasound generation, a dichroic absorber can allow both ultrasound generation and excitation laser delivery for photoacoustic imaging in one fiber. Due to narrow but strong absorption band, dye crystal violet (CV) and AuNPs were explored for hybrid transmitters [33]. Performance characteristics of various all-optical ultrasound transmitters are compared in Table 4. By changing the wavelength of laser source, both ultrasound generation from the absorbers and photoacoustic excitation at the target tissue were achieved. With a fiber-based FP sensor, both ultrasound and photoacoustic images of the same tissue were obtained with high resolution (Figure 10). Ultrasound imaging provide high penetration depth up to 15 mm, while photoacoustic imaging highlighted fatty tissue [33]. This design allows the hybrid imaging using only two optical fibers, which is promising to be further minimized. The collinear design also provides high sensitivity for intravascular imaging. With miniature size, high resolution and imaging depth, all-optical ultrasound, and photoacoustic imaging devices are promising to be used for clinical intravascular imaging. Besides FP etalon-based sensors, a microring was also used to fabricate an all-optical probe with images of phantoms achieved. The design of the probe is shown in Figure 11. Parameters of different all-optical imaging devices are shown in Table 4.

## 5. Summary and Future Prospects

In this work, we reviewed recent development on optical ultrasound generation and detection. With the use of nanomaterials such as CNTs and AuNPs, ultrasound signals with comparable intensity and frequency band width to conventional piezoelectric ultrasound transducers were achieved. Optical ultrasound sensor, such as fiber-based FP etalons, microring, and FBGs, also provided comparable sensitivity and bandwidth. Particularly, the planoconcave FP sensor exhibits ultrahigh sensitivity and at a wide range of detection angles. With high resolution and large penetration depth, all-optical IVUS/IVPA provides great promise for intravascular imaging. Using specific composite, such as dye CV and AuNPs-PDMS, hybrid transmitters were fabricated for both ultrasound generation and laser delivery through the same optical fiber. This advancement allows the two-fiber design for IVUS/IVPA imaging.

**Figure 12:**
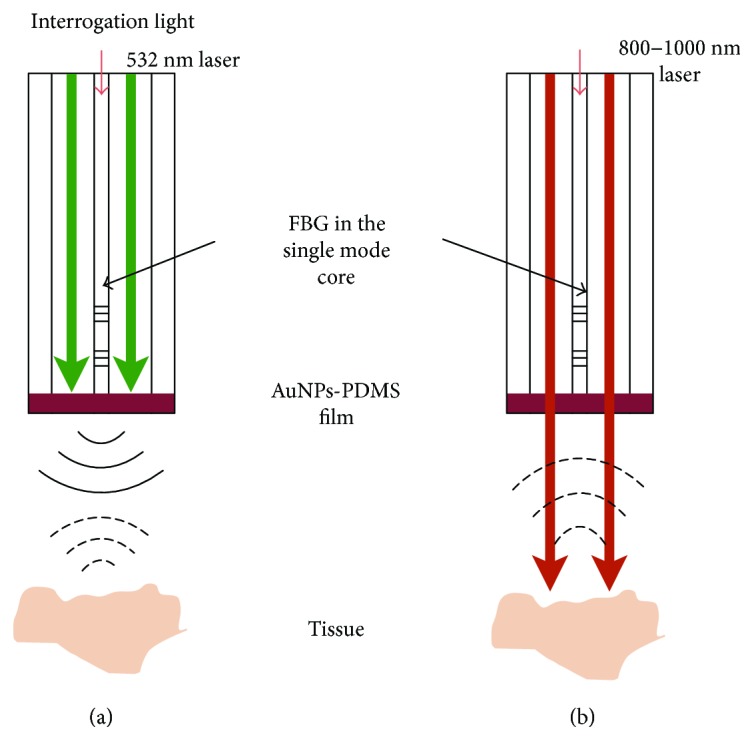
Schematic diagrams of an ultrasound and photoacoustic dual-modality imaging probe. (a) Ultrasound imaging mode. (b) Photoacoustic imaging mode.

In order to further improve the performance of all-optical imaging, works in three main aspects are required: firstly, the use of laser with varying pulse width for ultrasound generation and photoacoustic excitation needs to be studied. As the generated ultrasound frequencies depend on the pulse width of the excitation light, and higher frequency acoustic waves offer finer spatial resolution, but suffer from greater acoustic attenuation in biological tissue, the use of excitation light with different pulse widths is promising to provide both high resolution and large penetration depth in IVUS. Furthermore, since different tissue exhibit unique optical absorption spectra, using excitation light at multiple wavelengths could highlight a variety of tissue structures such as hemoglobin and lipid distributions [11, 69]. Secondly, while 20 frames per second was achieved recently [9], a higher speed is desired for clinical imaging. This limitation is mainly caused by the repetition rate of laser devices. Finally, further minimization is desired for intravascular applications. Dye CV and AuNPs-PDMS [33] enable the design of hybrid transmitters for both ultrasound generation and photoacoustic excitation. By coating these materials onto the double-cladding FP or FBG sensors, single fiber-based probe is promising to be achieved for IVUS/IVPA imaging. The design of the single fiber and FBG-based probe is shown in Figure 12 AuNPs-PDMS film generates ultrasound using 532 nm laser and allows the transmission of 800–1000 nm laser for photoacoustic excitation. Back-scattered ultrasound and photoacoustic signals are received by the FBG detector. As FBG can detect sideway ultrasound, both forward and sideway imaging are promising to be achieved with sideway ultrasound transmitter.

## Figures and Tables

**Table 1:** Summary of performance characteristics of planar optical ultrasound transmitters.

Absorber	Layer structure	Thickness (*μ*m)	Pressure (MPa)	−6 dB bandwidth (MHz)	Laser power (mJ/cm^2^)	Measurement distance (mm)
Carbon black [24]	Single-layer	25	0.15	~40	10.4	10
Carbon black [25]	Single-layer	11	0.8	~20	—	—
AuNPs [34]	Single-layer	80–1000	0.01–0.189	~4	3.67–13	1.8
CNTs [28]	Multilayer	2.6	—	~80	3 mW/cm^2^	1.4
CNFs [31]	Multilayer	57.9	12.15	7.63	3.71	3.65
rGO [27]	Multilayer	11.1	0.5	~60	35.66	—
AuNP Array [32]	Multilayer	~4.5	0.5	~65	50.9	10

**Table 2:** Summary of performance characteristics of fiber-based optical ultrasound transmitters.

Absorber	Layer structure	Thickness (*μ*m)	Fiber diameter	Pressure (MPa)	−6 dB bandwidth (MHz)	Laser power (mJ/cm^2^)	Measurement distance (mm)
CNTs [29]	Single-layer	—	105	0.45	12	41.6	2
CNTs [29]	Single-layer	—	200	0.9	15	36.3	2
AuNPs [35]	Single-layer	105	400	0.64	~8	8.75	~1
AuNPs [33]	Single-layer	~100	—	0.41	15.1	55.3	1.5
Dye [33]	Single-layer	~50	—	0.9	4.5	86.3	1.5
CNTs [30]	Multilayer	~20	200	1.34–4.5	23.15–39.8	16.2–87.9	3

**Table 3:** Summary of performance characteristics of optical ultrasound detectors.

Sensor	Geometry	Detection direction	Diameter (*μ*m)	Sensitivity (kPa)	Bandwidth (MHz)
FP etalon [36]	Cylindric cavity	Forward	6–10	15	20
FP etalon [37]	Concave cavity	Forward	125	0.4	20
FP etalon [38]	Dual cladding	Forward	—	—	—
FP etalon [38]	Dual cladding	Sideway	—	—	—
FP etalon [39]	Planoconcave cavity	Forward	5.2	0.093	40
Microring [41]	Ring	Forward	100	0.23	75
Microring [40]	Ring	Sideway	~800	0.352	250
FBG [42]	Cylindric grating	Sideway	-/(270 in length)	0.44	10
FBG [43]	Cylindric grating	Forward	-/(1500 in length)	9 n*ε*/Hz^1/2^	—

Noise equivalent pressure (NEP), the acoustic pressure with which signal-to-noise ratio equals 1, is used as the criterion to evaluate detectors. Forward refers to the axial direction of the optical fiber while sideway refers to the radial direction.

**Table 4:** Summary of performance characteristics of all-optical ultrasound and photoacoustic imaging systems in literature.

Imaging function	Transmitter	Diameter (mm)	Axial resolution (*μ*m)	Lateral resolution (*μ*m)	Imaging depth (mm)	Tissue imaging
Ultrasound [44]	CNTs-PDMS	0.84	64	88	3.5	Yes
Ultrasound [30]	CNTs-PDMS	—	—	—	12	Yes
Ultrasound [46]	CNTs-PDMS	2.5	—	—	9	Yes
Ultrasound [39]	CNTs-PDMS	—	65.9	94.2	—	Yes
Ultrasound [47]	Carbon black	3.5	110	97	0.9	Yes
Photoacoustic [39]	External source	—	—	36	—	Yes
Photoacoustic [41]	Optical fiber	4.5	—	—	—	No
Hybrid [67]	Carbon black-PDMS/optical fiber	~2	—	104–154 (photoacoustic) 64–112 (ultrasound)	4	No
Hybrid [33]	AuNPs-PDMS	—	—	—	15	Yes
Hybrid [33]	CV-PDMS	—	—	—	15	Yes
